# Xymedon Activates the Immune Response in Breast Cancer Xenografts

**DOI:** 10.3390/biomedicines13122996

**Published:** 2025-12-06

**Authors:** Alfiya Fakrieva, Ivan Raginov, Oxana Bondar, Peresvet Pets, Kirill Kryshen, Ramis Shabaev, Pavel Starokon, Konstantin Balakin

**Affiliations:** 1 Office of Technology Leadership, Kazan State Medical University, 420012 Kazan, Russia; 2 Institute of Fundamental Medicine and Biology, Kazan Federal University, 420008 Kazan, Russia; raginovi@mail.ru; 3 Department of General Pathology, Kazan State Medical University, 420012 Kazan, Russia; 4 Pathology Department, The Republican Clinical Hospital of the Ministry of Health of the Republic of Tatarstan, 420064 Kazan, Russia; 5 Scientific and Educational Center of Pharmaceutics, Kazan Federal University, 420008 Kazan, Russia; oxanav.bondar@gmail.com; 6 Research-and-Manufacturing Company “Home of Pharmacy”, 188663 Kuzmolovsky, Russia; peresvet-pez@mail.ru (P.P.); kryshen.kl@doclinika.ru (K.K.); 7 Department of Emergency and General Surgery Named After Professor A. S. Ermolov, Russian Medical Academy of Continuous Professional Education of the Ministry of Healthcare of the Russian Federation, 125993 Moscow, Russia; kampramis@mail.ru; 8 Department of Surgery with a Course in Oncology and Radiation Diagnostics, Branch of the Federal State Budgetary Educational Institution of Higher Education “S.M. Kirov Military Medical Academy” of the Ministry of Defense of Russia, 107392 Moscow, Russia; oldhorse.pm@mail.ru

**Keywords:** breast cancer, xenograft model, pyrimidine derivative, immunomodulation, Xymedon

## Abstract

**Background/Objectives:** Breast cancer remains a major cause of cancer-related mortality among women worldwide, highlighting the need for new therapeutic strategies. Pyrimidine derivatives have shown promise in oncology due to their ability to modulate immune responses and influence tumor growth pathways. **Methods:** Cytotoxicity of Xymedon was evaluated using MTT and colony formation assays on cancer MCF-7, NCI-H322M, HCT-15 cells, and primary human foreskin fibroblasts. In vivo efficacy was assessed in an orthotopic MCF-7 xenograft model in female Balb/c nude mice. Xymedon was administered orally at 410 mg/kg daily alone or in combination with intraperitoneal doxorubicin (1 mg/kg weekly). Hematological, histological, and immunohistochemical analyses were performed. **Results:** In vitro, Xymedon (up to 3 mM) showed no cytotoxicity against cancer cell lines or human skin fibroblasts. In vivo, Xymedon significantly increased tumor necrosis (44.1% vs. 28.5%, *p* < 0.01) and enhanced intratumoral infiltration of CD3+, CD8+, and CD20+ lymphocytes, with peritumoral counts increasing 2.2–5.3-fold. It mitigated Doxorubicin-induced myelosuppression by improving red blood cell counts, hemoglobin, and hematocrit levels, while platelet recovery remained limited. Combination therapy with Xymedon did not affect tumor volume or weight, but resulted in a non-significant trend toward improved survival (80% vs. 30%, *p* ≈ 0.11; Hazard Ratio [HR] = 0.268, 95% CI: 0.07082 to 1.012) without affecting fibrous capsule formation. **Conclusions:** These results suggest that Xymedon is a non-cytotoxic immunomodulator with potential as an adjuvant to enhance antitumor immunity and reduce hematologic toxicity associated with chemotherapy. Further studies are needed to elucidate the molecular pathways and confirm clinical efficacy.

## 1. Introduction

Breast cancer remains the second leading cause of cancer-related mortality among women worldwide, representing a major burden on health systems [1]. Pyrimidine derivatives have emerged as promising agents in cancer therapy due to their ability to modulate immune responses and target specific pathways involved in tumor growth and progression. These compounds exhibit a wide range of biological activities, including antiproliferative, pro-apoptotic, and immunogenic effects, making them valuable candidates for enhancing existing breast cancer therapies. The mechanism of action of pyrimidine derivatives involves multiple pathways, including the inhibition of key enzymes involved in nucleotide synthesis, modulation of immune checkpoints, and induction of immunogenic cell death. Critically, classical pyrimidine analogs (e.g., 5-fluorouracil) exert antiproliferative effects through disruption of DNA synthesis—either by inhibiting DNA polymerase or incorporating into nascent DNA strands, causing chain termination and replication stress [2]. For instance, JH530, a pyrimidinediamine derivative, has been shown to induce methuosis in TNBC cells by disrupting cellular homeostasis and triggering a non-apoptotic form of cell death and leading to tumor regression in xenograft models [3]. Additionally, TPH104, a thienopyrimidine analog, has been found to activate dendritic cells (DCs) and enhance the production of pro-inflammatory cytokines, such as TNF-α and IL-6, thereby promoting an immunogenic tumor microenvironment [4].

Pyrimidine compounds, integral to nucleotide biosynthesis, have been extensively studied for their therapeutic potential in oncology and immunology, with early investigations demonstrating their capacity to enhance NK cell cytotoxicity and T cell responses [5,6]. Over the past decades, advances in understanding pyrimidine metabolism and its inhibitors, such as dihydroorotate dehydrogenase (DHODH) blockers, have revealed their dual role in tumor suppression and immune modulation [7,8].

Pyrimidine derivatives exert their anticancer effects through diverse mechanisms, including protein kinase inhibition (crucial for cell growth and metabolism) and interference with nucleotide metabolism. By targeting these enzymes, pyrimidine compounds can effectively disrupt cancer cell proliferation and survival [9]. Fused pyrimidine derivatives, such as pyrazolo [3,4-d]pyrimidine and pyrido [2,3-d]pyrimidine, have also been explored for their anticancer potential. These compounds act as bioisosteres of purines, further expanding the range of pyrimidine-based therapeutics [9].

Some pyrimidine derivatives have been designed to selectively inhibit mutated forms of the epidermal growth factor receptor (EGFR), which are often implicated in cancer progression. This selective inhibition allows for targeted therapy, reducing the impact on normal cells and minimizing side effects [10,11]. In HER2-positive breast cancer, pyrimidine derivatives have been explored for their potential to sensitize tumor cells to HER2-targeted therapies. For instance, the combination of DNA methyltransferase (DNMT) and histone deacetylase (HDAC) inhibitors with anti-programmed death-ligand 1 (anti-PD-L1) antibodies has been shown to induce viral mimicry in breast cancer cells, leading to the activation of the retinoic acid-inducible gene I (RIG-I)–mitochondrial antiviral-signaling protein (MAVS) pathway and the production of type I interferons [12]. This immunomodulatory effect may enhance the antitumor activity of HER2-targeted therapies by promoting a pro-inflammatory tumor microenvironment.

Additionally, pyrimidine derivatives have shown potential in modulating immune responses. For instance, 2-(4-(2,5-dimethyl-1 H-pyrrol-1-yl)− 1 H-pyrazol-3-yl)pyridine can influence the expression of cytokines (upregulating cytokines such as IL4, IL6, and IL12/23p40 and downregulating INFγ in human PBMCs), thereby enhancing the body’s immune response against cancer cells [13]. The pyrimidinone compound, bropirimine, when administered to MCMV infected mice was able to restore mitogen-induced proliferative and IL-2 responses of splenic cells, increase the number of cells expressing Thy-1 or L3T4, and restore the ratio of T helper/T suppressor cells [5].

Pyrimidine derivatives act through the modulation of key enzymes such as DHODH and uridine-cytidine kinase 2 (UCK2), influencing nucleotide availability critical for lymphocyte proliferation and effector functions [7,14]. Concurrently, these compounds affect cytokine production, receptor expression, and signaling cascades in NK- and T-cells, thereby shaping immune responses [15]. Understanding these interrelations provides a foundation for dissecting the immunomodulatory mechanisms of pyrimidine derivatives.

Unlike classical pyrimidine analogs such as 5-fluorouracil, which exhibit antiproliferative activity through disruption of DNA synthesis, Xymedon (1-(2-hydroxyethyl)-4,6-dimethylpyrimidin-2-one) demonstrates predominantly immunomodulatory and reparative properties without direct cytotoxicity [16,17]. Xymedon stimulates the differentiation of T-lymphocyte precursors and increases both the total content of CD3+ cells and the CD4+ lymphocyte subpopulation, indicating its potential as an immunoadjuvant in antitumor therapy [18]. Xymedon’s immunomodulatory effects are mediated through multiple mechanisms, including stimulation of mitochondrial respiratory chain enzymes, reduction of adenylate cyclase activity, inhibition of Ca2+-ATPase activity, and increased intracellular DNA synthesis in thymocytes [19]. These mechanisms collectively contribute to enhanced T-lymphocyte maturation and function. Xymedon specifically induces the expression of CD2+ and DR+ molecules on immunocompetent cells, stimulates the migration of CD2+ lymphocyte subpopulations, and increases the affinity of CD2+ receptors [19]. Preclinical studies have demonstrated its ability to mitigate chemotherapy-induced toxicity while increasing CD4+ and CD8+ lymphocyte populations, suggesting immunomodulatory properties [18,20]. The primary objective of this study was to investigate the correlation between tumor growth dynamics and immune cell infiltration under Xymedon treatment to elucidate its dual role in chemosensitization and immune activation.

## 2. Materials and Methods

### 2.1. Study Protocol and Ethical Considerations

Prior to the initiation of the study, a detailed study protocol was prepared. This document outlined the primary research objectives, the experimental design (including animal housing conditions, quarantine procedures, and the definition of humane endpoints), and the pre-specified plan for data analysis. The protocol has not been formally registered in a public repository but is available from the corresponding author upon request.

All animal experiments were approved by the Bioethics Committee of the Research and Manufacturing company “Home of Pharmacy” (protocol No. 1.8/24) (Kuzmolovsky, Russia) and were conducted in accordance with the NIH Guide for the Care and Use of Laboratory Animals and standard operating procedures. The following humane endpoints were defined in the study for unscheduled euthanasia: prolonged immobility, lethargy, a moribund state, dyspnea, and a body weight loss of ≥20% from baseline.

### 2.2. Cell Culture, Compound

Human breast adenocarcinoma cells MCF-7, non-small cell lung carcinoma cells NCI-H322M, and colorectal adenocarcinoma cells HCT-15 were obtained from the American Type Culture Collection (ATCC). Primary human foreskin fibroblast cells (HSF) were isolated from the skin explant according to conventional protocol [21]. Cancer cells and HSF cells were cultured in DMEM and α-MEM media (PanEco, Moscow, Russia), respectively, supplemented with 10% fetal bovine serum (HyClone, Logan, UT, USA), L-glutamine and 1X penicillin-streptomycin (50 IU/mL/50 µg/mL) (PanEko, Moscow, Russia).

Xymedon (1-(2-hydroxyethyl)-4,6-dimethylpyrimidin-2-one), as an active pharmaceutical substance for in vitro studies and as a medicinal product for in vivo studies, was provided by “Tatchempharmpreparaty” JSC (Kazan, Russia). According to the instructions for medical use, the highest therapeutic dose (HTD) of Xymedon^®^ is 500 mg 4 times a day orally for a maximum course of up to 1 month (depending on the indication for use), or 2000 mg/60 kg, i.e., 33.3 mg/kg. The HTD for mice was calculated using standard conversion principles ≈ 410 mg/kg [22].

### 2.3. MTT-Assay

Cancer cells and human skin fibroblast (HSF) cells were seeded in 96-well plates with 2 × 10^3^ and 4 × 10^3^ cells per well, respectively. After 24 h, the cells were treated with Xymedon for 72 h at a concentration of 0.017–3000 µM. The medium was replaced with growth medium (80 μL), then 20 μL of MTT (5 mg/L) was added to each well, and the plate was incubated for 2 h at 37 °C. The culture medium was then removed, and 100 μL of DMSO was added to each well for 10 min. Absorbance at OD 555/700 was measured using a TECAN Infinite 200 PRO microplate reader (Grödig, Austria). Relative cell viability was calculated as a percentage of absorbance of the treated samples relative to the control. All experiments were performed in three independent biological replicates.

### 2.4. Colony Formation Assay

Cancer cells and human skin fibroblast (HSF) cells were seeded in 24-well plates at a density of 3 × 10^2^ and 4.5 × 10^2^ cells per well, respectively. After 24 h, cells were treated with Xymedon for 7 days at a concentration of 3 mM. After incubation, the colonies were washed twice with cold DPBS and fixed in fixation buffer (glacial acetic acid, methanol, distilled water in a ratio of 10:10:80). The colonies were then stained with 0.4% alcoholic crystal violet solution, then the colonies were lysed in 10% acetic acid and the absorbance at OD590 was measured using a TECAN Infinite 200 PRO microplate reader (Grödig, Austria). The results were presented as a percentage of the control values (untreated cells). All experiments were performed in three independent biological replicates.

### 2.5. Xenograft Animal Model

Female specific pathogen free Balb/c nude mice (8–10 weeks old, *n* = 65) were obtained from the Laboratory Animals Genetic collections Center (LAGCC) (Nizhny Novgorod, Russia). Upon arrival at the animal housing facility of RMC “HOME OF PHARMACY” JSC, the animals were placed in quarantine. The quarantine period lasted for at least 5 days. Prior to the start of the study, the animals were housed in individually ventilated cages.

To minimize potential confounders, treatment allocation and cage placement were randomized, and all experimental procedures were performed according to standard operation procedures and study protocol. Veterinarians, histology and pathology department staff, and clinical diagnostics department personnel were blinded to group details throughout the entire study.

Two days before MCF-7 cell transplantation, 60 mice were assigned numbers ranging from 1 to 60 and implanted subcutaneously (in the withers) with a tablet containing 17β-estradiol valerate (1.0 mg) and cholesterol (24.0 mg). On the 26th day of the experiment, Balb/c nude mice were additionally injected with a solution of 17β-estradiol valerate in a mixture of Matrigel and DMSO. The injection volume per mouse was 60 μL:56.67 μL of Matrigel and 3.33 μL of dimethyl sulfoxide; the dose of 17β-estradiol valerate was 0.22 mg per mouse.

Orthotopic xenografts were prepared by injecting 5 × 10^6^ cells mixed with 100 µL of Matrigel (Corning Life Sciences, Tewksbury, MA, USA) into the fat pad of the second thoracic mammary gland. Following tumor cell injection, primary tumor volume was measured 3 times per week using a digital caliper and a modified ellipsoidal formula (Volume = [length × width^2^]/2), where length was the longest axis and width was the measurement at right angles to length.

### 2.6. Study Design and Animal Allocation

Animal allocation into experimental groups was carried out using the matched pairs method. Group nos. 2–3 were formed when the tumor volume reached 150 mm^3^ or more, with a minimum of 20 mice meeting this criterion. Group nos. 4–5 were subsequently formed from the remaining animals after the formation of groups nos. 1–3.

In addition to the primary inclusion criterion for assignment to Groups 1 and 2 (a tumor volume of at least 150 mm^3^), animals were excluded during randomization based on the following secondary criteria:

(1) Presence of multiple neoplasms;
(2) Poor general clinical condition, characterized by severe prostration and significant body weight loss;
(3) A complex tumor morphology that precluded reliable volumetric measurement and could have substantially compromised the accuracy of size monitoring.

According to these criteria, 5 groups of animals (55 animals in total) were formed:

Group 1 (tumor-free control mice, *n* = 5) received starch per os by gavage (10 mL/kg) once daily for 28 days and saline i.p. (10 mL/kg) weekly for 4 weeks.

Group 2 (DOX, *n* = 10) received starch per os by gavage (10 mL/kg) once daily for 28 days and doxorubicin 1 mg/kg i.p. (10 mL/kg) weekly for 4 weeks.

Group 3 (Xym + DOX, *n* = 10) received Xymedon 410 mg/kg per os by gavage (10 mL/kg) once daily for 28 days and doxorubicin 1 mg/kg i.p. (10 mL/kg) weekly for 4 weeks.

Group 4 (Starch, *n* = 15) received starch per os by gavage (10 mL/kg) once daily for 5 days prior the implantation and 14 days after that.

Group 5 (Xym, *n* = 15) received Xymedon 410 mg/kg per os by gavage (10 mL/kg) once daily for 5 days before and 14 days after implantation.

Randomization was performed using a computer-generated random number sequence. The allocation concealment was maintained by an independent researcher not involved in the experiments. Blinding was applied during caliper measurements, survival assessment, and histopathological evaluation. For IHC analysis, two independent researchers performed the evaluation blinded to the group assignment, with an inter-rater agreement coefficient of 0.87 according to Spearman’s correlation.

Sample size determination for each experimental group was based on formal power analysis to ensure statistical rigor. For hematological parameter analysis, power calculations were performed using data from previously published literature. This analysis determined that a minimum of six animals per group would provide 80% statistical power to detect a 20% change in key hematological parameters (e.g., RBC count, hemoglobin concentration), with an assumed standard deviation of 10% and a significance threshold (α) of 0.05. For the implant contracture study (Groups 4–5), the initial group size (*n* = 10) was established based on methodological considerations from a comparable published investigation. To accommodate potential postoperative mortality and ensure adequate statistical power, an additional five animals per group were included, yielding a final group size of *n* = 15. Regarding the overall survival analysis, these data represent the first reported findings of this nature in the literature. Given that survival assessment was designated as an exploratory endpoint in our experimental design, a formal a priori power calculation was not conducted for this specific outcome measure.

### 2.7. Surgical Technique for Removal of Orthotopic Xenograft and Implantation of a Breast Implant Simulator

After treatment, radical surgical removal of the orthotopic xenograft of the mammary gland was performed with simultaneous installation of a breast implant simulator (d = 6 mm, SILTEX™ Round Breast Implants, High Profile, Cohesive I™, MemoryGel™, MENTOR^®^, Leiden, Netherlands).

Breast implant simulators were prepared by cutting out a section of the implant shell with a punch biopsy device (6 mm diameter) and scraping off the remaining silicone from the inner surface to avoid possible artifacts resulting from the passage of silicone mass into the capsule around the implant. Implant simulators were sterilized using 70% ethanol for 30 min and then air-dried for 1 h in a sterilized cell culture hood, as previously described 21.

Mice were anesthetized with a combination (mixture in one syringe) of zolazepam 10 mg/kg (Virbac, France) and xylazine 20 mg/kg (Alfasan International B.V., Woerden, Netherlands) intramuscularly. To confirm the effectiveness of anesthesia, the mice were tested for the absence of a withdrawal reflex when the paw was pinched.

At a distance of 2 mm to the right of the growing tumor, a skin incision of approximately 6 mm in length was made using curved surgical scissors. The skin was then turned outward. In our study, no adhesion of the tumor node to the underlying tissues was observed, so we performed their dissection using a blunt method. In case of bleeding, bipolar coagulation with the high-frequency medical electrosurgical device “Trilox-80” was used as a hemostasis method. Xenografts were fixed in 10% neutral buffered formalin for at least 24 h and embedded in paraffin.

After the tumor was removed, a silicone implant simulator was installed. The surgical wound was closed with PGA USP 3/0 suture material (BALF, Saint Petersburg, Russia). Additionally, BF-6 glue was applied on top in 3 layers to prevent postoperative complications. After the surgery, the animals underwent pain therapy: immediately after the intervention, the mice were given a single injection of tramadol intramuscularly at a dose of 10 mg/kg, then on the 2nd and 3rd days after the surgery, flexoprofen was given intramuscularly at a dose of 5 mg/kg with an interval of 24 h. To prevent dehydration, the mice were given a single injection of 0.5 mL of warm saline subcutaneously after the surgery.

On the first day after surgery, the animals were kept in a clean and dry cage with an absorbent diaper. The mice were observed daily. There were no cases of suture failure. Animals that showed severe general clinical condition, prolonged immobility, lethargy, dyspnea, weight loss ≥ 20% of the initial weight were subjected to unexpected euthanasia. These animals were considered to have died during the study.

Animals from groups 4–5 were euthanized on the 15th day after the implantation surgery. The installed silicone pieces were removed as a single block with all surrounding tissues, including a skin flap to orient the material for further histological examination, then fixed in 10% neutral buffered formalin for at least 24 h and embedded in paraffin.

Animals from groups 1–3 were euthanized on the 30th day after the start of test drug and vehicle administration. Xenografts, normal liver, heart, spleen, and kidney were surgically excised, then fixed in 10% neutral buffered formalin for at least 24 h and embedded in paraffin. Blood samples were collected in tubes with EDTA and analyzed within 1 h of collection using a Mythic 18 analyzer. The animals were not deprived of food or water prior to euthanasia and blood collection.

The following method of euthanasia was used: CO_2_ exposure was used at the initial stage of euthanasia, and exsanguination from the heart cavities was performed at the final stage of euthanasia.

### 2.8. Histology

Sections 3–5 μm thick were stained with hematoxylin and eosin. Silicone pieces were additionally stained with three-color histochemical staining according to Masson. Histological preparations were analyzed using a Mikromed-2 light-optical microscope (Micromed LLC, Saint Petersburg, Russia) at 40-, 100-, 200-, and 400-fold magnification. Microphotography was obtained using a TOUPCAM UCMOS05100KPA digital camera and ToupView 3.7.7892 software (China), which was also used to obtain panoramic images. Morphometric analysis was performed using the VideoTest Morphology software, Version 4.0 (VideoTest, Saint Petersburg, Russia) using photographs taken at a resolution of 200×.

For quantification of necrosis degree, we calculated acellular regions (appearing pale pink) within tumors as identified by H&E stain (area of tumor necrosis), and a cellular tumor region is defined as a hypercellular region (area of tumor).

**Figure 1:**
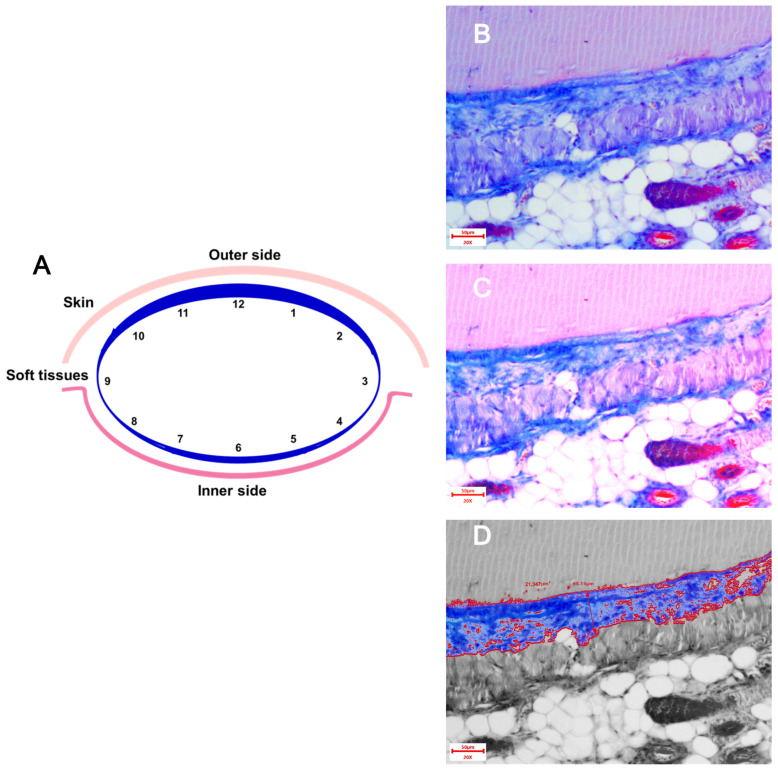
(**A**) Schematic diagram of the measurement of the thickness and area of the capsule. (**B**–**D**) Sections of the capsule wall around the implant stained according to Masson. Collagen fibers are stained blue. Magnification 200×. (**B**) Unprocessed image. (**C**) Image with enhanced contrast, saturation and brightness. It is evident that the blue color is more pronounced. (**D**) Processed image: blue color is left, the rest is black and white. The thickness and area of the capsule are shown.

The following morphometric parameters of the paraprosthetic capsule were assessed:

- Thickness of the connective tissue capsule in μm at 200×. The stained area of each implant was conditionally divided into three zones: “top—middle—bottom”, and in each zone, the capsule thickness was measured using the software in 4 fields of view. Thus, 12 measurements were obtained from each implant, for which the average value was calculated (Figure 1).
- Collagen fiber area in µm^2^ at 200×. Changing the brightness and contrast settings when photographing made it possible to differentiate the colors and, after image processing in the ToupView 3.7.7892 software, measure the area of blue staining in the field of view (Figure 1). The fields of view were selected similarly to the method for assessing the capsule thickness. The areas in 12 fields were summed up.

### 2.9. Immunohistochemical Staining

Immunohistochemical staining was performed on formalin-fixed paraffin-embedded tissue sections to assess the expression of CD3, CD8, and CD20 markers in cancer tissues.

The protocol included the following steps:

1. Slides were deparaffinized in xylene and rehydrated through a graded alcohol series.
2. Heat-induced antigen retrieval was performed by incubating slides in 0.01 M citrate buffer (pH 6.0) at 97 °C for 15 min in a water bath, followed by gradual cooling.
3. Endogenous peroxidase activity was quenched with 3% hydrogen peroxide.
4. Non-specific binding was minimized by applying a 5% bovine serum albumin (BSA) solution in 1× phosphate-buffered saline (PBS).
5. Sections were stained with the following antibodies:
  - Anti-CD3 (Servicebio, Wuhan, China, dilution 1:100),
  - Anti-CD8 (Servicebio, Wuhan, China, dilution 1:100),
  - Anti-CD20 (FineTest, Wuhan, China, dilution 1:100).
6. After incubation of primary antibodies, slides were treated with biotinylated secondary antibodies (Novocastra, conjugated with horseradish peroxidase) for 1 h, and then visualized using 3,3′-diaminobenzidine (DAB) chromogen.
7. Sections were counterstained with hematoxylin and fixed permanently.

The number of CD3-, CD8-, CD20-positive cells was determined in at least five high-power fields per section at 400× magnification. Results were expressed as the mean number of positive cells per high-power field across five fields, averaged per tumor. All assessments were performed independently and blinded by two investigators with an inter-rater agreement coefficient of 0.87 (Spearman correlation). Intratumoral regions were defined as areas containing tumor cells with minimal stromal components, while peritumoral regions were defined as the interface between tumor tissue and surrounding normal tissue (within a 500 μm distance from the tumor margin).

### 2.10. Statistical Analysis

All quantitative data were initially screened for outliers using Grubbs’ test. The normality of data distribution was assessed with the Shapiro–Wilk test. Depending on the distribution, data are presented as the mean (M) ± standard deviation (SD) for normally distributed parameters, or mean (M) with 95% CI or as the median (Me) and interquartile range (Q1;Q3) for non-normally distributed parameters. For the comparison of two independent groups, either the two-tailed Student’s *t*-test or the Mann–Whitney U test was applied, based on the data distribution. Comparisons among three or more groups for non-normally distributed quantitative data were conducted using the Kruskal–Wallis test, followed by a post hoc test for multiple comparisons of mean ranks. The dynamics of tumor growth and body weight were analyzed using two-way ANOVA with the Geisser–Greenhouse correction. Overall survival data were visualized with Kaplan–Meier curves, and the curves were compared using the log-rank test. Statistical analysis was performed using licensed software: Statistica 10 (StatSoft, Tulsa, OK, USA) or GraphPad Prism 9.1.1 (GraphPad Software, San Diego, CA, USA). Differences were considered statistically significant at *p* < 0.05.

The primary data (in both electronic and paper formats), as well as the complete study archive, are deposited and available for review at the premises of RMC “HOME OF PHARMACY” JSC.

## 3. Results

### 3.1. Effect of Xymedon on Cell Growth

**Figure 2:**
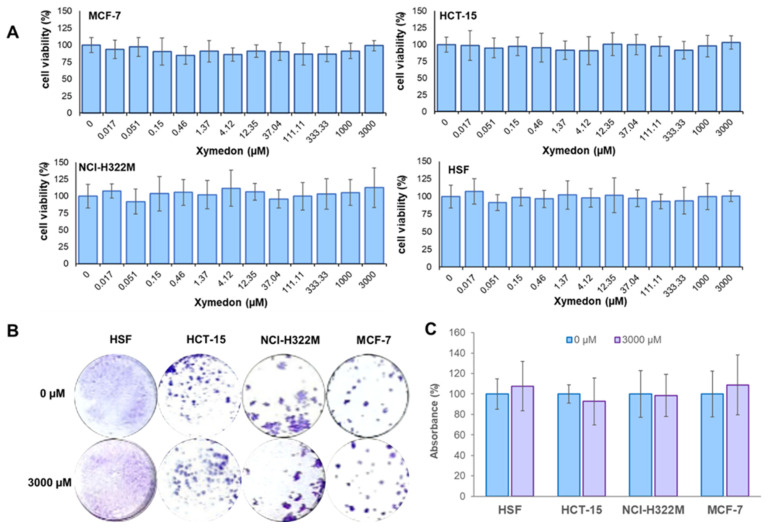
Effect of Xymedon on cell growth. (**A**) MTT assay. Cells were treated with 0–3000 µM Xymedon for 72 h. Results are presented as a percentage of control. (**B**) Representative images of colony formation, and (**C**) quantitative analysis of the absorbance at OD590 of cell lysates of colonies formed by cells treated with 0–3000 µM Xymedon for 7 days. Results are presented as a percentage of control. Numbers are representative of three independent experiments.

The effect of Xymedon on cell growth was analyzed using MTT and colony formation assays. The results are shown in Figure 2.

The results of this experiment show that Xymedon (1.4–3000 μM) does not exert statistically significant inhibitory or stimulatory effects on the proliferation of the human tumor cell lines studied (MCF-7, NCI-H322M, HCT-15) as well as conditionally normal human skin fibroblast (HSF) cells. The IC_50_ values for all cell lines exceeded 3000 µM.

### 3.2. In Vivo Studies

#### 3.2.1. Effect of Xymedon and Doxorubicin on Tumor Growth In Vivo

**Figure 3:**
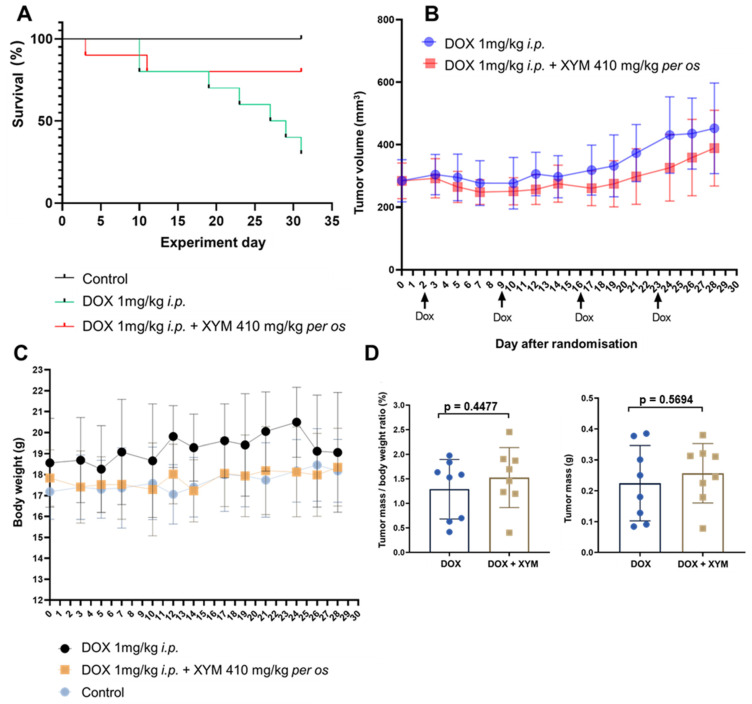
Effect of Xymedon and Doxorubicin on tumor growth in vivo. (**A**) Kaplan–Meier survival plot. Effect of DOX and DOX + XYM on (**B**) tumor volume; (**C**) body weight; (**D**) tumor weight to body weight ratio. Data from animals that died between days 3 and 11 after randomization were not included in the analysis. Data from the remaining animals were included in the analysis. The mice were divided into three groups. Each animal from the control group received starch p.o. by gavage (10 mL/kg) once daily for 28 days and saline i.p. (10 mL/kg) weekly for 4 weeks (control). Each animal from the experimental groups received starch p.o. by gavage (10 mL/kg) once daily for 28 days and doxorubicin 1 mg/kg i.p. (10 mL/kg) weekly for 4 weeks (DOX group) or Xymedon 410 mg/kg p.o. by gavage (10 mL/kg) once daily for 28 days and doxorubicin 1 mg/kg i.p. (10 mL/kg) weekly for 4 weeks (DOX + XYM group). The results are presented as the mean ±SE.

Following randomization and initiation of doxorubicin chemotherapy, a subset of mice exhibited signs of worsening clinical condition, leading to premature deaths. Survival outcomes were compared using the log-rank test and are presented in the Kaplan–Meier plot (Figure 3A). Doxorubicin monotherapy significantly reduced survival compared to the control group (*p* < 0.05). However, the addition of Xymedon to doxorubicin (DOX + XYM group) did not confer a statistically significant survival benefit over doxorubicin alone (*p* > 0.05; Hazard Ratio [HR] = 0.268, 95% CI: 0.07082 to 1.012). Although statistical differences were not found between the DOX and DOX + XYM groups, a non-significant trend towards increased overall survival was noted in the group of animals receiving Xymedon during chemotherapy (80% vs. 30%, *p* ≈ 0.109).

The kinetics of tumor growth during treatment are shown in Figure 3B. Xymedon did not significantly alter the tumor growth rate, as two-way ANOVA with Geisser–Greenhouse correction found no significant effect of the “group” factor (*p* > 0.05). Upon planned euthanasia on day 31, tumors were excised and weighed. The final tumor mass and the tumor mass to body mass ratio were comparable between the DOX and DOX + XYM groups (Figure 3D), with no statistically significant differences revealed by a two-sided Student’s *t*-test (*p* > 0.05). On average, tumors reached a mass of 220–250 mg, representing 1–1.5% of the total body mass at the endpoint.

**Figure 4:**
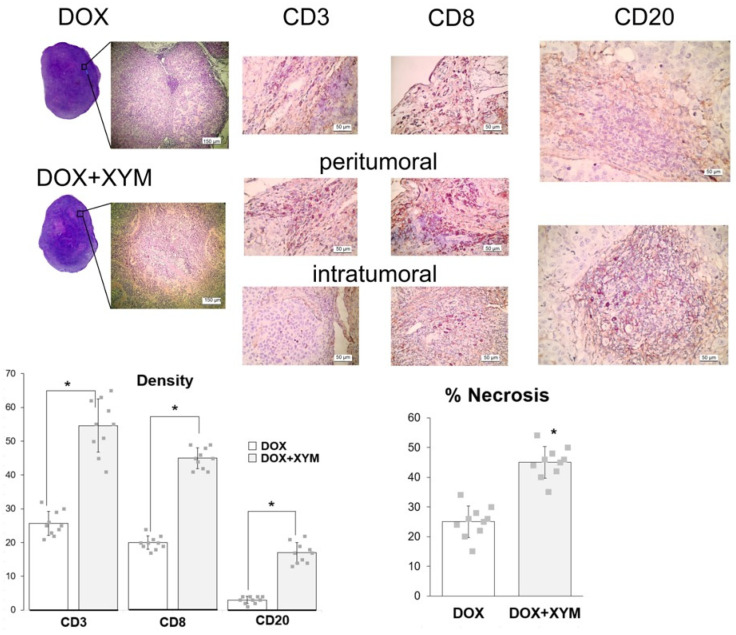
H&E staining and immunohistochemistry (IHC) imaging of CD3+ T cells, CD8+ T cells, and CD20+ B cells. *—significant differences between DOX and DOX + XYM groups, *p* ˂ 0.05. The results are presented as the mean ±SD.

Thus, the study confirmed that the introduction of Xymedon during doxorubicin chemotherapy does not affect the growth dynamics of breast cancer (human ductal breast adenocarcinoma MCF-7). Quantification of the area of necrosis on H&E sections revealed that DOX + XYM tumors had significantly more necrosis compared to DOX tumors (44.1 ± 12.1% vs. 28.5 ± 8.8%, respectively, *p* < 0.01) (Figure 4).

In the DOX group, CD3+-, CD8+-, and CD20+-lymphocytes were located only in the peritumoral areas. Under the influence of Xymedon, CD3+-, CD8+-, and CD20+-lymphocytes were detected both in the peritumoral areas and among tumor cells (intratumorally). When comparing the number of CD3+-, CD8+-, and CD20+-lymphocytes in the peritumoral areas, a significant increase in these cells in the XYM + DOX group was shown by 2.2, 2.3, and 5.3 times, respectively.

#### 3.2.2. Hematology

Data on hematological parameters and the results of statistical processing are presented in Table 1. Data on animals sacrificed before planned euthanasia that received less than four injections of doxorubicin were excluded from processing.

According to the obtained data, Xymedon statistically significantly increased the concentration of red blood cells and hemoglobin compared to the group of animals receiving doxorubicin alone, which led to an increase in hematocrit. Thus, Xymedon contributed to an increase in the formed elements of blood, which are reduced by oncology and chemotherapy. These findings suggest that Xymedon may partially counteract doxorubicin-induced myelosuppression in erythropoiesis, potentially through antioxidant mechanisms protecting hematopoietic stem cells from oxidative stress. However, the platelet count (PLT) remained reduced in both groups (DOX: 297 × 10^9^/L; DOX + XYM: 379 × 10^9^/L) without significant differences between them, which emphasizes the limited efficacy of the drug in restoring megakaryocytic hematopoiesis. Analysis of leukocyte parameters showed that doxorubicin caused pronounced lymphopenia (LYM%: 82.10% vs. 91.50% in the intact group). However, Xymedon partially increased the absolute number of lymphocytes (LYM × 10^9^/L: 2.10 vs. 1.70 in the DOX group). At the same time, an increased proportion of monocytes (MON%: 8.00% vs. 5.80% in the DOX group) and neutrophils (GRA%: 11.60% vs. 11.90% in the DOX group) was observed, which may reflect the selective stimulating effect of the drug on myeloid hematopoiesis.

#### 3.2.3. Effect of Xymedon Alone on Tumor Growth Before Implantation

**Figure 5:**
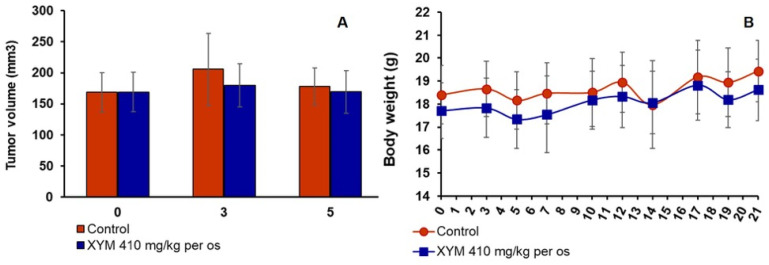
Effect of Xymedon on (**A**) tumor volume and (**B**) body weight. Mice received Xymedon 410 mg/kg per os by gavage (10 mL/kg) once daily for 5 days prior the implantation. Results are presented as the mean ± SE.

Xymedon, when administered per os for 5 days, had no effect on tumor volume or mouse weight (Figure 5).

**Figure 6:**
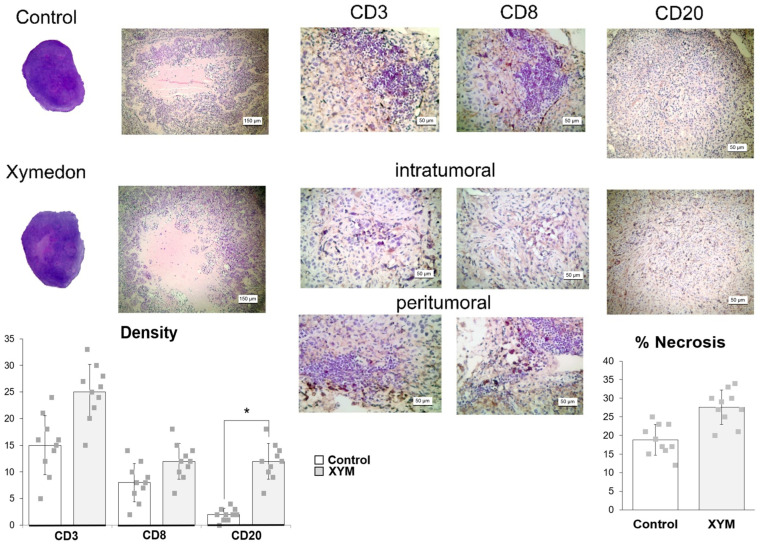
H&E staining and immunohistochemistry (IHC) imaging of CD3+ T cells, CD8+ T cells, and CD20+ B cells. *—significant differences between Control and XYM groups, *p* < 0.05. The results are presented as the mean ± SD.

As in the previous series of experiments, immunohistochemistry staining (IHC) for CD3+ and CD8+ T cells showed that they were excluded from the central areas of control tumors and were restricted to the periphery (petitumoral) (Figure 6).

In contrast, tumors in the Xymedon group contained increased numbers of CD3+ and CD8+ T-cells and CD20+ B-cells but there was a significant difference only for CD20+ cells (Xymedon −11.8 ± 2.1% vs. Control −2.4 ± 0.8%; (*p* < 0.01).

#### 3.2.4. Effect of Xymedon on Capsule Thickness and ECM Formation

**Figure 7:**
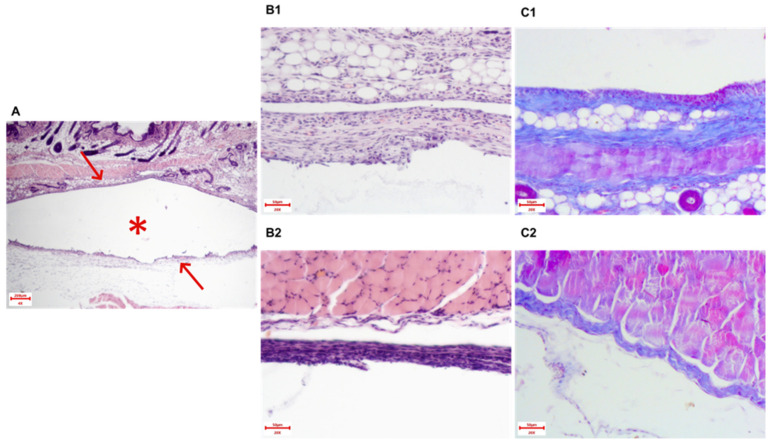
Section of the capsule wall around the implant. (**A**) The capsule wall (arrows) with well-formed structured connective tissue fibers, * cavity formed by the implant. Hematoxylin and eosin staining, magnification 40×. (**B**) Section of the capsule wall around the implant, in the bed (bottom) area. Capsule wall with well-formed structured connective tissue fibers. Magnification 200× (**B1**) Hematoxylin and eosin staining, (**B2**) Masson’s trichrome staining. (**C**) Section of the capsule wall around the implant, outer part. Capsule wall with well-formed structured connective tissue fibers; moderate amount of lymphocytes, macrophages and many fibroblasts are present. Magnification 200× (**C1**) Hematoxylin and eosin staining, (**C2**) Masson’s trichrome staining. The scale bar in A is 200 μm, and the scale bars in B1, B2, C1, and C2 are 50 μm.

According to the necropsy results, all mice from the Xymedon group showed 100% healing of postoperative wounds and no implant exposure at the time of planned euthanasia. Five animals died before planned euthanasia without further histology analysis. In the animals studied, the material was represented by a skin flap with underlying soft tissues containing a cavity surrounded by a dense fibrous capsule (Figure 7A).

The capsule is well formed, presented as a thin, well-defined connective tissue structure when stained with Masson’s trichrome, completely surrounding the implant. In the area of the bottom (bed), the capsule wall is thin, adjacent to the muscles, the inflammatory reaction is extremely weak (Figure 7(B1,B2)). Outwardly, the wall thickens and is most pronounced directly under the skin (Figure 7(C1,C2)). In addition, from the surface, a more pronounced tissue reaction to a foreign object was observed in the form of moderate infiltration by lymphocytes, macrophages and fibroblasts, but signs of acute or purulent inflammation were absent, and the formation of connective tissue was also well expressed.

**Figure 8:**
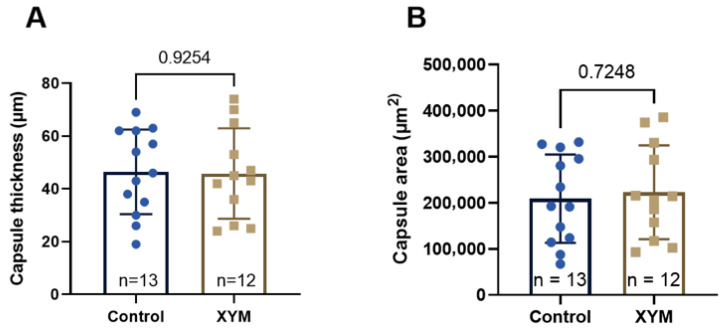
Results of the assessment of the thickness (**A**) and area (**B**) of the paraprosthetic capsule. The data obeyed the law of normal distribution (Shapiro–Wilk criterion). Together with individual values, the graph presents the mean value (M), standard error of the mean (SD), and the number of animals in groups (*n*). Two-sided Student’s *t*-test for independent variables did not reveal a statistical difference between the thickness (**A**) and area (**B**) of the capsule in the control and Xymedon groups.

The histological analysis included assessment of the capsule thickness and fibrous tissue area. The results of the quantitative assessment are presented in Figure 8. The capsule thickness in groups 3–4 averaged 46 μm, with a total area in twelve visual fields of ~200 μm^2^.

Data analysis did not reveal statistical differences (Student’s *t*-test, *p* > 0.05) in the thickness of the fibrous capsule and its area in animals. Also, no effect of Xymedon administration (5 days before and 14 days after surgery at a dose of 410 mg/kg) on the process of formation of the fibrous capsule around the mammary gland prosthesis installed after radical removal of the MCF-7 tumor was found.

## 4. Discussion

In the present study, the effects of Xymedon, a pyrimidine derivative, on tumor growth dynamics, immune cell infiltration, and hematological parameters were investigated in a mouse model of breast cancer. These results are consistent with the growing interest in pyrimidine-based compounds as multitarget anticancer agents, particularly due to their ability to modulate protein kinase activity, reverse multidrug resistance (MDR), and influence immune responses [23,24,25]. Our results revealed that while Xymedon lacks direct antitumor or antiproliferative activity, it possesses a distinct immunomodulatory potential.

In vitro assays demonstrated that Xymedon concentrations up to 3 mM did not inhibit the proliferation of MCF-7, NCI-H322M, HCT-15 cancer cells, or human skin fibroblasts (HSF). This is in contrast to previous studies on other pyrimidine derivatives such as PP242, which exhibit dose-dependent antiproliferative effects via ATP-competitive inhibition of mTOR [26]. Similarly, in vivo experiments showed no significant differences in tumor volume or weight groups receiving doxorubicin with or without Xymedon. However, histological analysis revealed a marked increase in necrotic areas in Xymedon-treated tumors (44.1% vs. 28.5% in the control group), suggesting an indirect effect on tumor viability. This necrosis may be caused by increased immune-mediated cytotoxicity or altered metabolic stress, although further mechanistic studies are needed [27].

While the difference in overall survival between the DOX and DOX + XYM groups did not reach the conventional threshold for statistical significance (*p* > 0.05), the substantial hazard ratio (HR = 0.268, 95% CI: 0.07082 to 1.012) suggests a biological signal that warrants discussion. Mortality in experimental animals during tumor progression results from multiple systemic factors, including systemic toxicity, doxorubicin-induced cardiotoxicity, systemic inflammatory response, and other tumor-related complications. Consequently, it is probable that Xymedon enhanced animal survival without affecting tumor size by suppressing systemic disturbances induced by the tumor. The difference in overall survival between the DOX + XYM and DOX groups was 80% vs. 30%, respectively (Figure 3A). The lack of statistical significance in the survival analysis (*p* ≈ 0.11) limits definitive conclusions but suggests a biologically relevant effect that merits further investigation with larger sample sizes and longer follow-up periods The improved survival without significant impact on tumor volume may be associated with several factors. First, Xymedon demonstrated the ability to enhance the immune response against the tumor, resulting in increased necrosis (44.1% vs. 28.5%, *p* < 0.01) and lymphocyte infiltration of the tumor. This may indicate suppression of the tumor’s metastatic potential, although direct data on metastasis were not collected in our study. Second, Xymedon partially mitigated the hematotoxic effects of doxorubicin, which could have improved the general condition of the animals. Third, we observed a trend toward reduced cardiotoxic effects of doxorubicin, which could also have influenced survival outcomes.

The study by Korzhova et al. demonstrated that the pyrimidine derivative SNK-578 (2-isobutyl-4,6-dimethyl-5-hydroxypyrimidine chlorohydrate), administered i.p. at a dose of 10 mg/kg on days 2–15, significantly increased the median survival of C57BL/6 mice with Lewis lung carcinoma from 28 days to 43 days, representing a 38.6% increase in lifespan [28]. In contrast, SNK-411 (2-isobutyl-4,6-dimethyl-5-hydroxypyrimidine) showed only a negligible reduction in tumor volume and did not significantly enhance survival compared to SNK-57827.

A key finding was Xymedon’s ability to promote intratumoral infiltration of CD3+, CD8+, and CD20+ lymphocytes, along with a 2.2- to 5.3-fold increase in peritumoral lymphocytes compared with doxorubicin monotherapy. This is consistent with preclinical data that pyrimidine derivatives can activate immune pathways such as the pyrimidinosome-mediated ferroptosis and metabolic regulation of T-cell function [27]. Although we did not examine the detailed molecular mechanisms of Xymedon’s action in this study, existing literature data suggest several potential pathways. Xymedon’s immunomodulatory effects are mediated through multiple mechanisms, including stimulation of mitochondrial respiratory chain enzymes, reduction in adenylate cyclase activity, inhibition of Ca2+-ATPase activity, and increased intracellular DNA synthesis in thymocytes [16,19,29,30]. These mechanisms collectively enhance T-cell survival and function, explaining the increased lymphocytic infiltration observed in our xenograft model. Previously, the ability of Xymedon to increase the number of lymphocytes was shown in various pathologies. Thus, the drug increased the levels of CD3+ cells and activated CD69 cells in bronchial asthma [16], and the levels of CD3+, CD4+ and CD8+ T-lymphocytes in chronic rheumatic heart disease [17].

After short-term administration of Xymedon (410 mg/kg, 5 days of treatment), we observed increases in tumor necrosis area (not significant), CD3 and CD8 cell counts (total and killer T-cells, respectively) (not significant), and CD20 cells (B-lymphocytes) (significant). With prolonged administration of Xymedon (410 mg/kg, once daily for 4 weeks), all of these parameters increased significantly. Importantly, without Xymedon, both T- and B-lymphocytes were detected only in the peritumoral space, while only under the influence of Xymedon did immune cells appear inside the tumors. For several tumor types, CD8+, CD4+, and CD20+ cell levels have been shown to positively correlate with oncologic prognosis [31]. Notably, the immunostimulatory effects of Xymedon contrast with the lack of direct cytotoxicity, highlighting its potential as an adjuvant to enhance tumor immunogenicity. Similar observations have been reported for other immunomodulatory agents such as JSI-124, which synergistically with tyrosine kinase inhibitors recruits immune cells to the tumor microenvironment [32].

While the Balb/c nude mouse model permitted successful engraftment of human breast cancer xenografts, its limitations for comprehensive immunomodulatory studies must be acknowledged. The observed T lymphocytes (CD3+, CD8+) and B lymphocytes (CD20+) exhibited functional constraints inherent to the nude phenotype. Consequently, our findings of enhanced lymphocyte infiltration should be interpreted as evidence of Xymedon’s capacity to modulate lymphocyte trafficking and/or survival within the tumor microenvironment, rather than as confirmation of a fully competent adaptive immune response. Definitive assessment of Xymedon’s immunomodulatory mechanisms, particularly those involving T-cell functionality, necessitates future investigation using immunocompetent syngeneic models.

Xymedon mitigated doxorubicin-induced myelosuppression by increasing red blood cell counts, hemoglobin levels, and hematocrit, potentially through antioxidant pathways or by preserving hematopoietic stem cells from oxidative stress. This is consistent with previous studies demonstrating the erythroprotective effects of Xymedone in rodent models [20]. It is important to note that we did not examine reticulocytes or bone marrow in our study, which limits our ability to fully explain the hematopoietic recovery mechanisms. Future research will include comprehensive hematopoietic progenitor analysis to better understand Xymedon’s mechanism of selective myeloprotection. However, platelet counts remained suppressed in both groups, indicating limited efficacy in restoring megakaryocytic hematopoiesis. This selective myeloprotective effect may reflect Xymedon’s preferential action on erythroid precursors or differential sensitivity of hematopoietic lineages to doxorubicin toxicity. Alternative explanations include the possibility that the doxorubicin dose used (1 mg/kg weekly) was particularly damaging to megakaryocytic progenitors, or that Xymedon’s mechanism of action primarily affects early erythroid differentiation rather than megakaryopoiesis. It is worth noting that in studies with rats bearing Walker-256 carcinoma treated with docetaxel, selective improvement in erythropoiesis was also observed with limited thrombocytopenia recovery [20]. Future studies should investigate dose–response relationships and include the assessment of bone marrow progenitor populations to clarify these mechanisms. The drug also partially reversed lymphopenia by increasing absolute lymphocyte counts with increased monocyte and neutrophil proportions, suggesting a shift toward myeloid differentiation. Such dual effects on erythropoiesis and myelopoiesis require further study, particularly with regard to cytokine signaling or preservation of the bone marrow niche [18].

Histological analysis of fibrous capsules around mammary implants revealed no differences in collagen thickness and area between the Xymedon-treated and control groups. This suggests that Xymedon does not increase foreign body reactions, a critical factor in patients undergoing cancer surgery. Previous studies have shown that agents such as tamoxifen reduce capsule formation via anti-inflammatory mechanisms [33], but the neutral effect of Xymedon in this work supports its safety profile in surgical contexts.

Compared to other immunomodulators, Xymedon’s profile is unique in its combination of myeloprotective and immunostimulatory effects without direct cytotoxicity. Unlike immunostimulants such as interferon or interleukin-2, which can cause significant toxicity [34], Xymedon appears to have a favorable safety profile. Its mechanism differs from checkpoint inhibitors which block inhibitory signals but require pre-existing tumor-infiltrating lymphocytes [19]. Instead, Xymedon appears to increase lymphocyte numbers and promote their infiltration into tumors, potentially creating a more favorable environment for checkpoint inhibitor therapy.

Overall, Xymedon demonstrates a dual role in counteracting chemotherapy-induced myelosuppression and promoting antitumor immunity without direct cytotoxic effects. These results position it as a promising adjuvant in breast cancer therapy, particularly to enhance the efficacy of immune checkpoint blockade or mitigate treatment-related toxicity. Further preclinical and clinical studies are needed to optimize its therapeutic application.

## 5. Conclusions

This study demonstrates that Xymedon, a pyrimidine derivative, exerts immunomodulatory and hematopoietic effects without direct antiproliferative activity against breast cancer cells in vitro or in vivo. Although Xymedon did not suppress tumor growth in combination with doxorubicin, it significantly enhanced tumor necrosis and promoted intratumoral infiltration of CD3+, CD8+, and CD20+ lymphocytes, which correlated with improved immune surveillance. These results highlight its role in modulating the tumor microenvironment to promote antitumor immunity, which is a critical factor in overcoming resistance to immunotherapy. Furthermore, Xymedon mitigated doxorubicin-induced myelosuppression by increasing red blood cell counts and hemoglobin levels, suggesting protective effects against chemotherapy-associated hematologic toxicity. In particular, its neutral effect on the formation of fibrous capsule around breast implants confirms its safety in postoperative oncology conditions.

The molecular mechanisms underlying Xymedon’s effects, as elucidated by Slabnov and colleagues, involve the stimulation of T-lymphocyte differentiation and protection against apoptotic processes in immune cells. These mechanisms provide a foundation for understanding its immunomodulatory properties observed in our study.

While the tendency toward improved survival did not reach statistical significance (*p* ≈ 0.11), the consistent pattern of enhanced immune infiltration and reduced hematologic toxicity suggests clinical relevance. The selective restoration of erythropoiesis but not megakaryopoiesis warrants further investigation to optimize dosing strategies or combination regimens.

Mechanistically, Xymedon’s effects appear to be distinct from pyrimidine-based protein kinase inhibitors, as it lacks direct cytotoxicity but enhances immune cell recruitment and metabolic resilience. These results position Xymedon as a promising adjuvant for breast cancer therapy, particularly in combination with immune checkpoint inhibitors or regimens targeting tumor immunogenicity. Future studies should investigate its effects in immunocompetent models, explore synergistic combinations with checkpoint inhibitors, and evaluate its impact on metastatic progression and long-term survival. This work is consistent with the growing paradigm of repurposing pyrimidine derivatives to bridge metabolic regulation, immune activation, and cancer therapy, with Xymedon representing a unique approach that enhances host immunity rather than directly targeting tumor cells.

## Figures and Tables

**Table 1:** Effect of Doxorubicin and Xymedon on hematological parameters. Data are presented as the median with 95% reference range (2.5th–97.5th percentile).

	Intact Group *n* = 5	DOX *n* = 3	DOX + XYM *n* = 8
WBC, 10^9^/L	5.8 (3.62–8.1)	1.90 (1.33–2.76) *	2.60 (1.88–4.18) *
LYM, %	91.5 (86.96–91.97)	82.10 (77.45–87.04)	80.20 (71.62–83.03) *
MON, %	3.9 (3.51–5.52)	5.80 (5.33–10.65)	8.00 (6.93–8.79) *
GRA, %	4.5 (3.77–7.64)	11.90 (7.15–12.57)	11.60 (9.76–25.48) *
LYM, 10^9^/L	5.2 (3.3–7.36)	1.70 (1.04–2.27) *	2.10 (1.43–2.30) *
MON, 10^9^/L	0.2 (0.11–0.49)	0.10 (0.10–0.10)	0.20 (0.12–0.39) **
GRA, 10^9^/L	0.3 (0.2–0.58)	0.20 (0.11–0.39)	0.30 (0.30–0.59)
LMR	25.5 (14.45–30.5)	17.00 (10.35–22.70)	10.50 (7.71–13.69) *
MLR	0.04 (0.03–0.07)	0.06 (0.04–0.10)	0.10 (0.07–0.13) *
LGR	15.5 (11.8–25.4)	5.75 (5.04–16.44)	7.00 (3.38–7.67) *
GLR	0.06 (0.04–0.08)	0.17 (0.06–0.20)	0.14 (0.13–0.30) *
RBC, 10^12^/L	7.39 (6.96–8.83)	7.16 (6.99–7.29)	8.16 (7.80–8.54) **
HGB, g/L	152.00 (144.1–172.8)	154.00 (147.35–156.85)	167.00 (158.75–171.70) **
HCT, %	0.33 (0.31–0.38)	0.34 (0.32–0.35)	0.37 (0.36–0.39) **
PLT, 10^9^/L	468.00 (221.9–611.3)	297.00 (295.10–333.10)	379.00 (324.80–543.95)

* significant differences relative to the intact group, *p* ˂ 0.05. ** significant differences between DOX and DOX + XYM groups, *p* ˂ 0.05

## Data Availability

The data are contained in the article.

## Abbreviations

The following abbreviations are used in this manuscript:

DOX	Doxorubicin
XYM	Xymedon
